# Age and gender effects on presence, user experience and usability in virtual environments–first insights

**DOI:** 10.1371/journal.pone.0283565

**Published:** 2023-03-27

**Authors:** Mario Lorenz, Jennifer Brade, Philipp Klimant, Christoph-E. Heyde, Niels Hammer

**Affiliations:** 1 Professorship for Production Systems and Processes, Chemnitz University of Technology, Chemnitz, Germany; 2 Department of Orthopedics, Trauma and Plastic Surgery, University Hospital Leipzig, Leipzig, Germany; 3 Division of Macroscopic and Clinical Anatomy, Gottfried Schatz Research Center, Medical University of Graz, Graz, Austria; 4 Division of Biomechatronics, Fraunhofer Institute for Machine Tools and Forming Technology IWU, Dresden, Germany; Central University of Finance and Economics, CHINA

## Abstract

Virtual Reality (VR) is applied in various areas were a high *User Experience* is essential. The sense of *Presence* while being in VR and its relation to *User Experience* therefore form crucial aspects, which are yet to be understood. This study aims at quantifying age and gender effects on this connection, involving 57 participants in VR, and performing a geocaching game using a mobile phone as experimental task to answer questionnaires measuring *Presence* (ITC-SOPI), *User Experience* (UEQ) and *Usability* (SUS). A higher *Presence* was found for the older participants, but there was no gender difference nor any interaction effects of age and gender. These findings are contractionary to preexisting limited work which has shown higher *Presence* for males and decreases of *Presence* with age. Four aspects discriminating this study from literature are discussed as explanations and as a starting point for future investigations into the topic. The results further showed higher ratings in favor of *User Experience* and lower ratings towards *Usability* for the older participants.

## 1 Introduction

Virtual Reality (VR) is a powerful tool with ubiquitous applications ranging from marketing [1–4], product development [5–7], and training [8–12] to even include rehabilitation [13–19] and therapeutic approaches [20–23]. As the primary purpose of VR is to support humans, it is of relevance to understanding human-related factors, especially for VR applications in medicine. Key aspects in this context are *User Experience* and *Usability*.

In the long history of defining *Presence*, there have been a number of definitions, which vary in concerning their scope [24]. Most definitions define *Presence* in the context of mediated environments, used to induce a feeling of ‘being there’ (see section 3.1.1 in [24]). As Skarbez et al. pointed out, this definition seems already suitable. However, ‘being there’ could be a more suitable description for *Place Illusion*, and would free the term *Presence* to describe what it is commonly used for: the general goodness of a virtual experience [24]. We agree with this reasoning and therefore, follow the definition of Skarbez et al. who defined *Presence* as “The perceived realness of a mediated or virtual experience.” [24]. This definition focuses on the subjective perception of the user and also includes the plausibly of the experience, which goes beyond the feeling of only ‘being there’ as a solely spatial sensation. According to the ISO9241-210 standard, *User Experience* is defined as “A person’s perceptions and responses that result from the use and/or anticipated use of a product, system or service.” [25] and *Usability* being defined as the “extent to which a system, product or service can be used by specified users to achieve specified goals with effectiveness, efficiency and satisfaction in a specified context of use” [25].

For VR training applications, a high *User Experience* is desirable to enhance the effects of the training [8, 9, 26–28]. A high *User Experience* and *Usability* further help to increase the acceptance of medical VR applications [29]. *User Experience* is furthermore important for product developers, where it has become a powerful instrument to assess the potential success of a product even before a physical prototype exists. VR can also create a more standardized assessing environment for field experience, whilst at the same time providing a more realistic experience than most other laboratory conditions. Established methods do exist to measure *User Experience*; however, these methods have been developed to be applied with real prototypes, not for virtual prototypes. Applying such methods in VR evaluation can therefore lead to erroneous conclusions and render the advantages of VR evaluations obsolete.

In a first baseline study, our team could show that VR evaluations on measures of *User Experience* and *Usability* may get affected by the *Presence* users have in VR [30]. Using a previously validated testing environment, which involved a five-sided CAVE, we aimed at assessing if such differences in age and/or gender exist with vast implications on the development of VR environments perceived as being real. It was unclear at this stage of the study if these effects were spread equally among the population investigated, which involved both sexes and a broad age range. There are anecdotal reports on *Presence*, *User Experience* and *Usability* having age- and gender-specific effects. Manifold studies have to date exclusively researched the effects of age on navigation and wayfinding skills, where VR is just used as an experimental environment [31–35]. Furthermore, for research on age related memory capacities, VR is used as an experimentation environment [36]. Gender influence in VR has been researched under the aspect of proximities to avatars [37] or the perception of embodied avatar hands in relation to their gender [38]. A meta-analysis of Peck et al. [39] discussed potential gender bias on simulator sickness and suggested researching whether such biases could be evident for other factors like *Presence*. Very few studies, however, investigate age and gender effects on *Presence* [40–42]. Additionally, no work so far has looked at both factors in one study. More data on gender and age-related effects on *Presence* is consequently and urgently needed. Moreover, as questionnaires on *Presence*, *User Experience* and *Usability* are widely established, a more detailed knowledge is necessary for practitioners to interpret their results from VR *User Experience* studies, and to prevent investigators from drawing false or biased conclusions. For the scientific community, the outcomes of such studies may be helpful to further refine such surveys, thereby making them usable and reliably in VR, or to find corrective values, improving their validity and to compensate for the bias introduced by VR.

Our group could show that connections exist between *Presence*, *User Experience* and *Usability* [30]. We could furthermore show that confounders exist for this connection on a cognitive level, and assessed the effects of low-level ethanol intake on these factors [43]. We decided to build upon these previous studies and further explore the existence of age- and gender-related effects on *User Experience* measures in a further experimental VR study.

We aimed at investigating the research question of whether any age- or gender-related differences in *Presence*, *User Experience* and *Usability* exist in VR. Main contributions of this given manuscript would be as follows:

- This study for the first time investigates age- and gender-effects on *Presence*; so far only one factor is investigated amongst existing studies.
- The influence of age and gender on *User Experience* and *Usability* of an application is for the first time evaluated deploying a virtual environment.
- Specific directions for future research regarding the effects of age and gender on *Presence* are provided.

## 2 Materials and methods

### 2.1 Experimental setup

Institutional approval for this study was obtained from the Institute for Machine Tools and Production Processes of the Chemnitz University of Technology. Ethical approval was obtained from the University of Leipzig (number: 251/17-ek), and all participants gave their informed written consent for their participation in the given study. Previous data from an earlier study, obtained under the same ethics protocol number, were also included [30, 43]. All experiments were conducted according to the principles of the Declaration of Helsinki.

**Fig 1:**
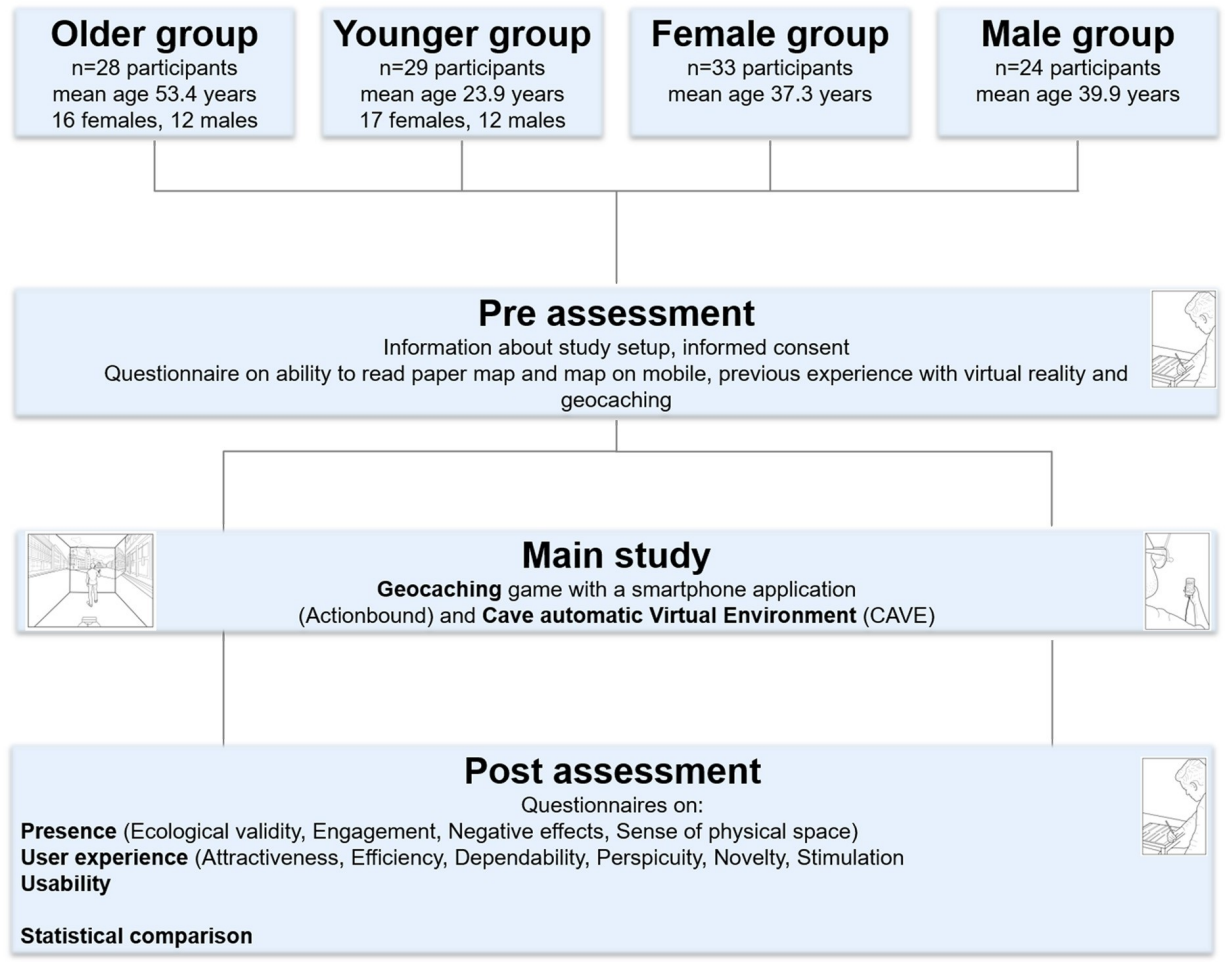
Graphical representation of the experimental procedure (Drawings by Robbie McPhee).

*Age* and *Gender* were used as independent variables as well as eleven dependent variables for *Presence*, *Usability* and *User Experience*. For *Gender*, participants were split into two groups: female and male [44]. For *Age*, the groups were defined as follows: 18–32 years of age (*Younger* group) and 48–62 years of age (*Older* group), providing a clear separation between the cohorts. Twenty-eight samples were included from a previous study on *Presence*, *User Experience* and *Usability* [30]. A further 29 participants were tested additionally. All participants were recruited using social media channels and mailing lists. To ensure comparability with our previous study [30], we applied an identical study protocol. This protocol was separated as follows: (1) pre-assessment, (2) main study and (3) post-assessment (see Fig 1). At the beginning of the pre-assessment, the participants were welcomed by the principal investigator and received verbal explanation detailing the task they would need to fulfill in the virtual environment. Following this introduction, the participants were asked to read and sign a form declaring their informed consent. For the next step, a demographic questionnaire was provided, asking information about age, gender and educational background, as well as the participants’ self-assessed ability to read digital and paper maps. Furthermore, the participants were asked if they had previous contact with VR and geocaching.

**Fig 2:**
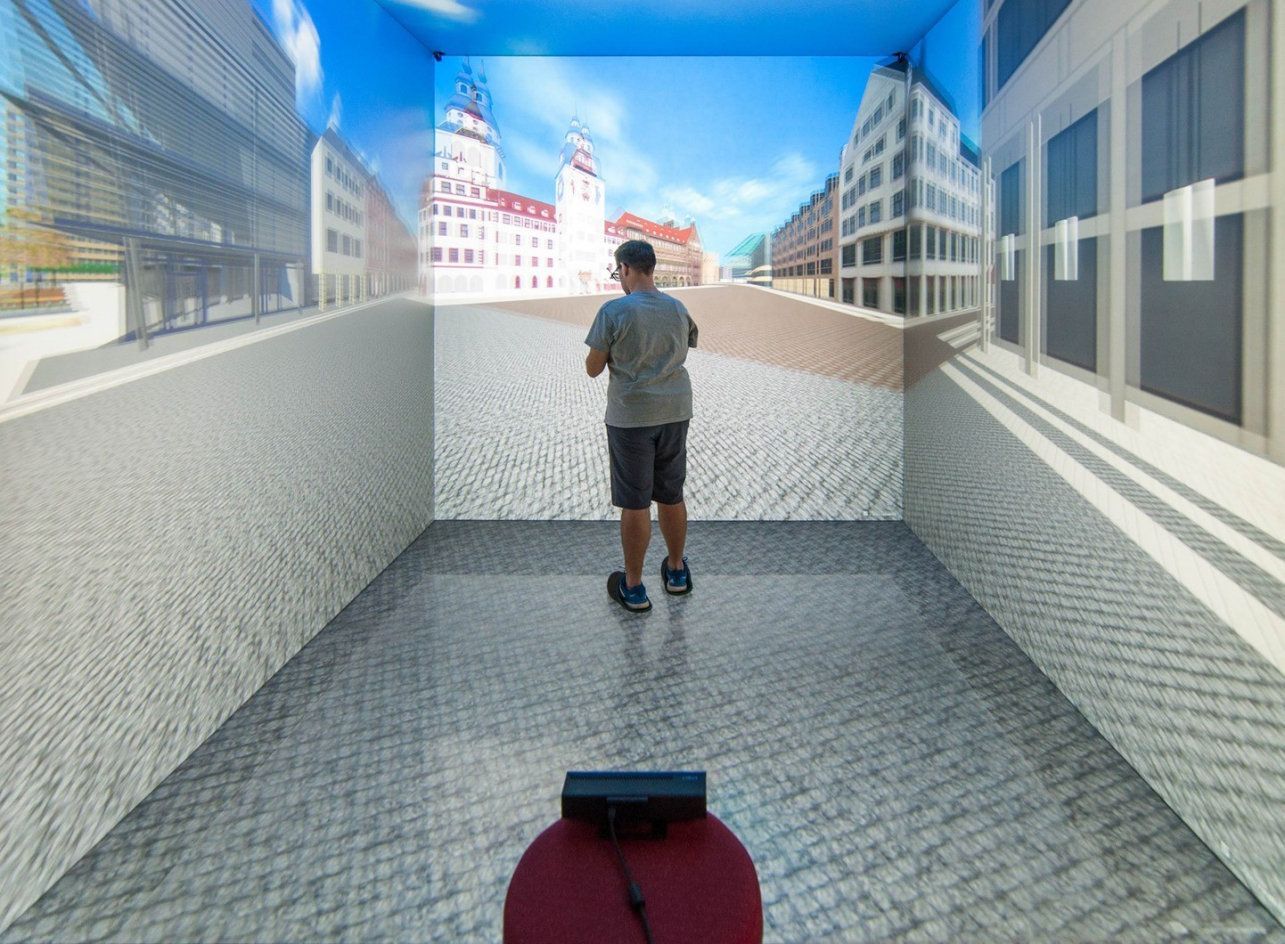
The virtual reality study setup showing the experimental setup in the virtual city center of Chemnitz, Germany inside the five-sided cave automatic virtual environment.

Upon completion of the demographic questionnaire, the main study began. The participants were guided to the virtual environment where they received further information about their objectives and the method of navigating within the virtual environment. The experimental task consisted of a geocaching game in the virtual city center of Chemnitz, Germany, using a smartphone application on a physical mobile phone. The geocaching game was implemented using the Actionbound application of Simon Zwick and Jonathan Rauprich GbR [45] and consisted of seven location with a tour length of 1.7 km. The participants were instructed by the information provided on the screen where their next target location within the virtual city center has been depicted. On the same screen, a 2D map of the city center was shown alongside the position of the users, to help provide information to orientate themselves. The user position was updated on the 2D map via an artificial Global Positioning System (GPS) signal, sent by the virtual environment to the mobile phone. Before the geocaching game started, each participant was asked to get familiar with the navigation method. The geocaching task was started once the participants informed the principle investigator that they felt comfortable with the navigation. A five-sided cave automatic virtual environment (CAVE) was used to immerse the participants into the VR scenario (see Fig 2). The CAVE had an edge length of 3 m and was built based on the principles of Cruz-Neira et al. [46, 47]. Twenty full HD rear projectors in combination with passive circular polarization enabled stereoscopic vision, and a cluster of eleven computers equipped with NVidia Quadro 6000 video cards rendered the virtual scene. Six optical infrared cameras by ART GmbH (Weilheim i. OB., Germany) tracked the participant’s head for calculating their viewpoint. For the navigation, a method developed by Lorenz et al. [48] was used, utilizing a Microsoft Kinect sensor, which tracked the movements of the participants from behind (see Fig 2). After finishing the main study, the participants concluded the post-assessment by answering questionnaires on *Presence* in the virtual environment as well as *User Experience* and *Usability* with the geocaching application. In a last step, the principal investigator debriefed the participants and answered questions about the study. Task completion time was not measured, as it was deemed irrelevant for the initial study design on the assessment of possible age- and gender- related effects on *Presence*, *User Experience* and *Usability*.

### 2.2 Dependent variables: *Presence*, *User Experience* and *Usability*

To measure *Presence*, a 12-item short form of ‘The International Test Commission–Sense of Presence Inventory’ (ITC-SOPI) by Lessiter et al. [49] was used. It consists of four scales: 1) Sense of Physical Space, 2) Engagement, 3) Ecological Validity and 4) Negative Effects, as applied previously for this purpose [30]. Each item was rated using a five-point Likert scale. The ITC-SOPI was chosen as it is intended for cross-media usage, which seemed most appropriated given the experiment includes the usage of a mobile phone.

*User Experience* [50] describes the subjective assessment of a product by a user. The User Experience questionnaire (UEQ) by Laugwitz et al. [51] was used in this study, and is a validated questionnaire to measure *User Experience*. On a seven-point sematic differential, 26 bipolar items are rated by the user. Out of these items, 6 scales were derived: 1) Attractiveness, indicating a positive or negative attitude towards a product; 2) Perspicuity, 3) Efficiency and 4) Dependability, which are summarized as the pragmatic aspects of a product; and 5) Stimulation and 6) Novelty, which represent the hedonic aspects of the product.

*Usability* assesses the users’ impression of the fitness of use of a product and corresponds to the pragmatic aspects of *User Experience*. A well-established questionnaire that was used in this study to assess *Usability* was the System Usability Scale (SUS) by Brook [52] where *Usability* is rated on a one-to-five Likert scale.

### 2.3 Statistical methods

A Kolmogorov-Smirnov-test was used to check the residues for normal distribution, followed by a Levene-Test to check for equality of error variances. A two-way multivariate analyses of variances (MANOVA) was performed thereafter to find possible age and gender related effects. P-values equal to or less than 0.05 were considered as statistically significant.

## 3 Results

### 3.1 Participant demographics

The age of the *Younger* group averaged 23.9 (SD = 2.8) years and was significantly younger (p < .001) then the average age of the *Older* group (M = 53.4, SD = 3.8). Participants of the *Younger* group were significantly more familiar with VR systems (20.7 %) compared to the *Older* group (0 %, p = 0.01). In the *Younger* group, 34.5 % of the participants were familiar with geocaching, which is significantly more frequent in comparison to the *Older* group (7.1 %; p = 0.01). The ability to read a paper map or a map on a mobile phone yielded no significant differences between the age groups (see Table 1).

**Table 1:** Distribution of participant age, and survey results of the participants’ self-assessment on their ability to read a map (i.e. paper or on a mobile phone), and on their previous contact with virtual reality systems and geocaching.

Group	Younger Group	Older Group	p-value
Mean age in years	23.9	53.4	0.001
	(SD = 2.8)	(SD = 3.8)	
Ability to read a paper map	Excellent = 24.1%	Excellent = 32.1%	0.76
	Good = 62.1%	Good = 50.0%	
Ability to read a map on a mobile	Excellent = 34.5%	Excellent = 35.7%	0.81
	Good = 51.7%	Good = 42.9%	
Previous contact with VR systems (yes)	20.7%	0%	0.01
Previous contact with geocaching (yes)	34.5%	7.1%	0.01

For statistical comparison between the *Younger* and *Older* groups, p-values are provided. SD = standard deviation.

The Female and the Male groups did not differ significantly in terms of age, their ability to read a map on a mobile phone nor on previous contact with VR systems. A significant difference was found for previous contact with geocaching (p = 0.046), with 30.3 % reported for the Female group and 8.3 % in the Male group. A further significant difference (p = 0.005) was found for the ability to read a paper map, with 72.6 % of the Female group reporting excellent or good abilities, in contrast to 100 % of the Male group (see Table 2).

**Table 2:** Distribution of participant age, and results of the participants’ self-assessment on their ability to read a map (i.e. paper or on a mobile phone), and on their previous contact with virtual reality systems and geocaching.

Group	Female Group	Male Group	p-value
Mean age	37.3	39.9	0.22
	(SD = 14.7)	(SD = 16.1)	
Ability to read a paper map	Excellent = 54.4%	Excellent = 58.3%	0.005
	Good = 18.2%	Good = 41.7%	
Ability to read a map on a mobile	Excellent = 42.4%	Excellent = 41.7%	0.08
	Good = 30.3%	Good = 54.2%	
Previous contact with VR systems (yes)	6.1%	16.7%	0.20
Previous contact with geocaching (yes)	30.3%	8.3%	0.046

For comparison between the Female and Male groups, p-values are provided. SD = standard deviation.

### 3.2 Age has a main effect but there are no interaction effects between age and gender

The results of the MANOVA in Table 3 show a significant main effect for age (p = 0.009) on the factors of the ITC-SOPI, UEQ and SUS questionnaires with a large effect size (Partial Eta-square of 0.411). In contrast, no gender or interaction effect of Age and Gender on *Presence*, *User Experience* and *Usability* were found.

**Table 3:** Results of the MANOVA using Pilai’s trace for age, gender, and the interaction effect of age and gender on *Presence*, *User Experience* and *Usability*.

	F ratio	df	df error	p-value	Partial Eta-square
Age (Older vs. Younger)	2.75	11.00	43.00	0.009	0.411
Gender (Male vs. Female)	1.25	11.00	43.00	0.284	0.243
Age/Gender	1.50	11.00	43.00	0.168	.277

df = degrees of freedom.

### 3.3 Presence is slightly higher for older, usability for younger, and user experience for older participants

**Fig 3:**
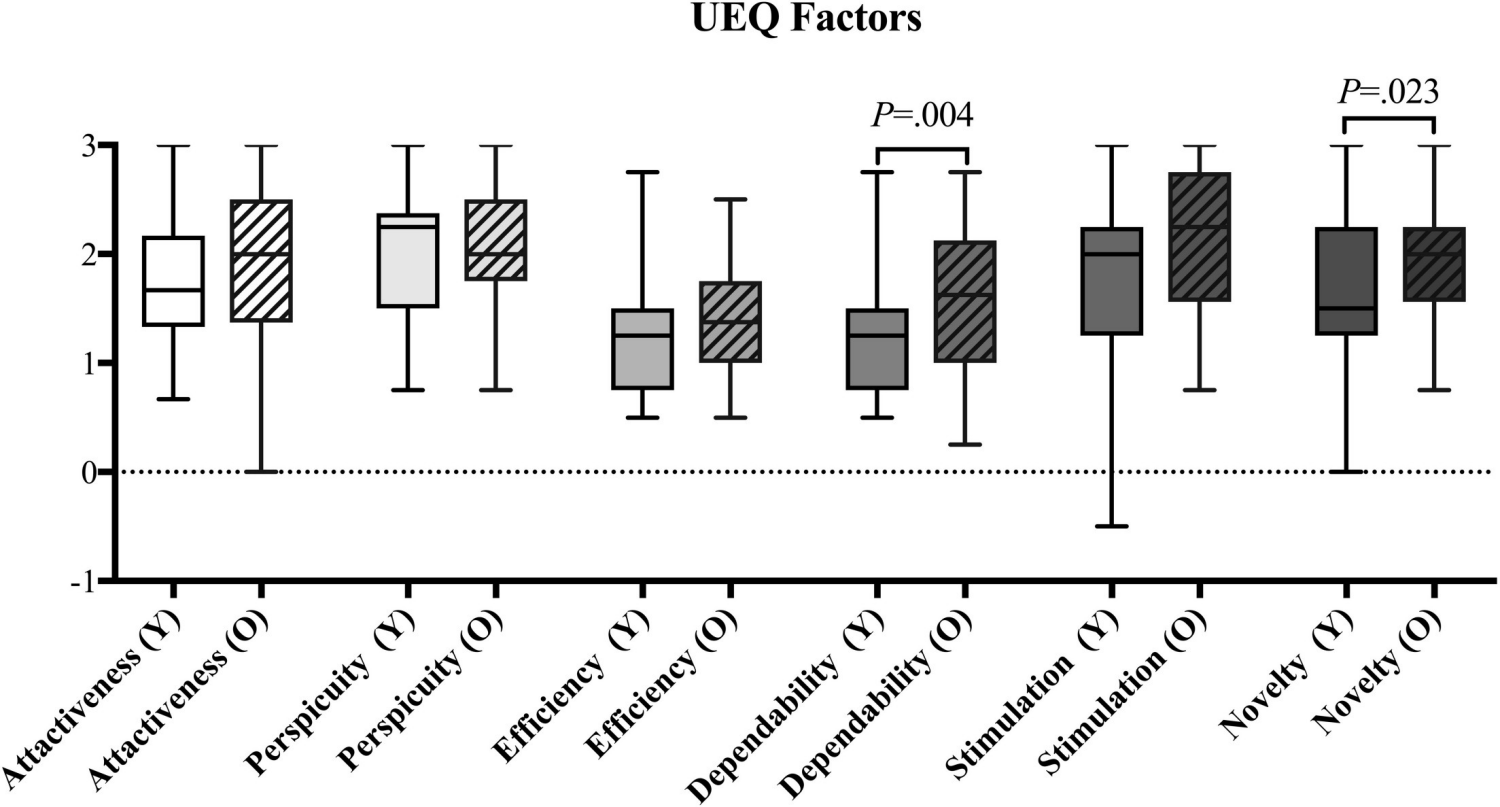
Boxplot of *User Experience* factors for the *Younger* group (Y), and for the *Older* group (O), showing large variability for most factors in both groups. All factors showed differences in the median, but only Dependability and Novelty showed differences on a significant level. Whiskers indicate the 25^th^ and 75^th^ percentiles.

**Fig 4:**
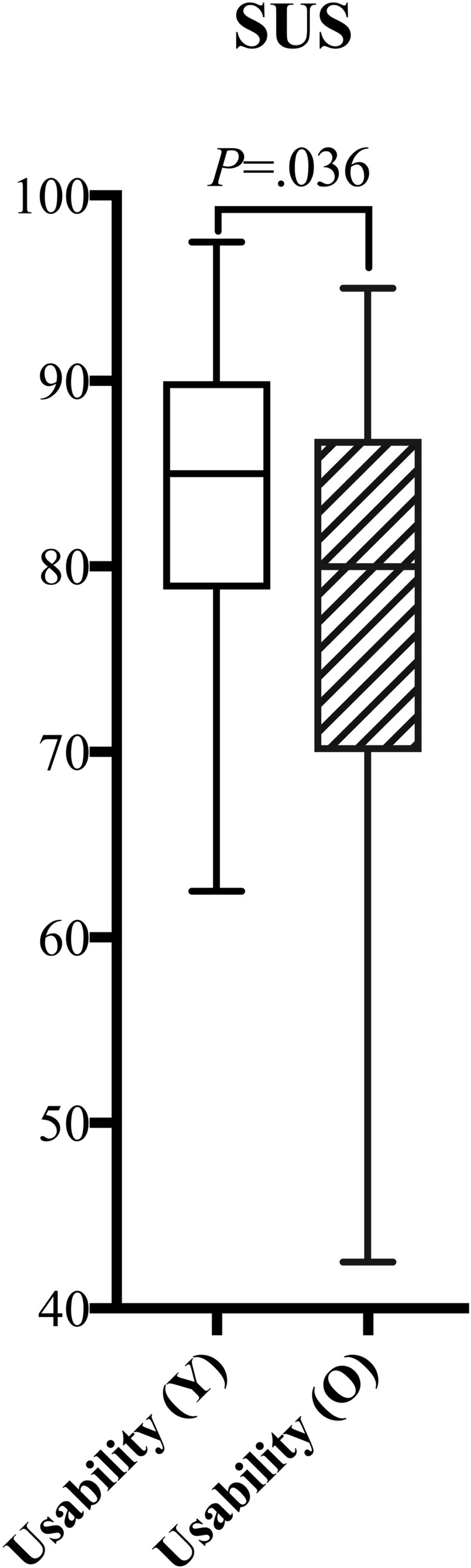
Boxplot of *Usability* for the *Younger* group (Y) and for the *Older* group (O), showing large variability in both groups and larger variability in the *Older* group. There was a significant difference in *Usability* showing higher values for the young. Whiskers indicate the 25^th^ and 75^th^ percentiles.

**Fig 5:**
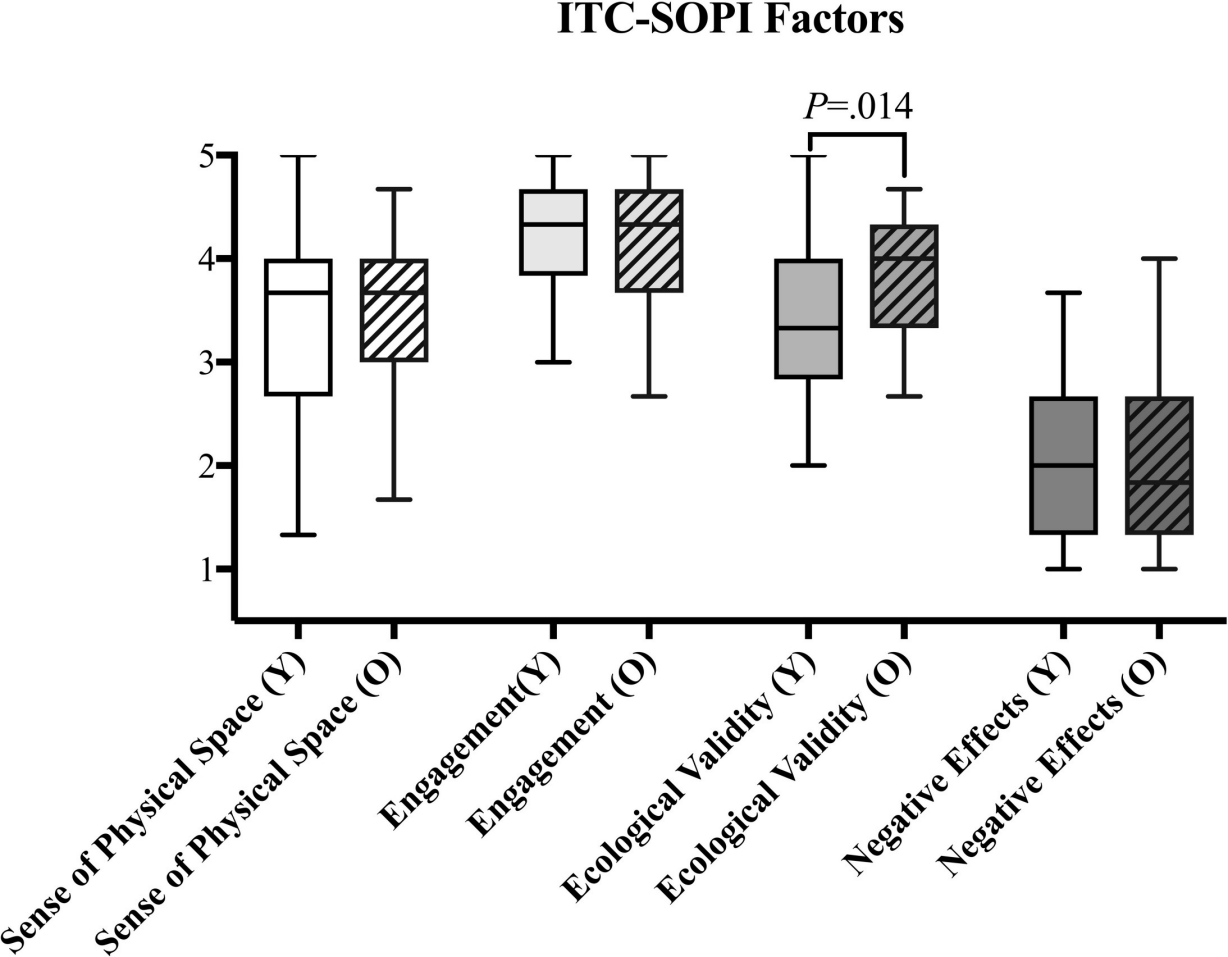
Boxplot of *Presence* factors for the *Younger* group (Y) and *Older* group (O), showing almost equally large variabilities in both groups and similar medians, except for ecological validity. This factor shows significantly larger differences in the medians and the variability of the groups being larger for the *Younger* group. Whiskers indicate the 25th and 75th percentiles.

It was shown that 5 of the 6 *User Experience* items were significantly higher in the *Older* group but for only two of these items (i.e. Dependability p = 0.004, Novelty p = 0.023) (see Fig 3). In contrast, *Usability* tended to be significantly higher for the *Younger* group (p = 0.036, see Fig 4), indicating that the younger participants found the application more efficient and reliable. No age-related differences were found for *Presence* except Ecological Validity (p = 0.014, see Fig 5), indicating that the virtual environment was more realistic and believable for older compared to younger participants.

## 4 Discussion

This study has underlined that *Presence* is only minimally affected by age and that no gender- and age-interaction effects appear to exist. In the literature, only the effects of age on navigation and wayfinding skills or on memory capacity has been researched, where VR is just used as an experimental environment [31–36]. As for researching gender bias in VR, only aspects like the proximities to avatars [37] or the perception of embodied avatar hands in relation to their gender [38] have been investigated. Only in a meta-analysis of Peck et al. [39] a potential gender bias on simulator sickness is discussed. Consequently, these studies cannot be used as a comparison to our results. Very few studies, however, investigate age and gender effects on *Presence* [40–42], for which a comparison to our results of the same can be made. Additionally, no work so far has looked at both factors in one study.

Literature investigating the effects of *Age* on *Presence* was so far mostly related to the younger population and to a less-well balanced male to female ratio [41]. Further, in one study presented by Kober [41], only one group used a VR setup, and for another group on the elderly, patients with brain lesions were included, forming a significant confounder as brain injury might impair or alter the mechanisms of how *Presence* is forming. All of the four studies in Kober [41] used different *Presence* questionnaires and sample sizes to the effect that the results are difficult to compare. Our study used a standardized setup with a larger sample size (n = 57) and a large age range amongst participants to specifically address these issues. Kober concluded that *Presence* declines with age. However, only the two groups with a smaller sample size (n = 20; n = 21) [41], showed significant effects, out of which one group (n = 21) used a non-VR setup and included brain-injured participants. In contrast, another two groups showed an increase of *Presence* with aging although not on a significant level. These contradictory results are worth further examination. Although for our analysis we used a multivariate analysis of variances instead of a regression analysis as Kober [41] did, we cannot report lower *Presence* ratings for the *Older* group. Rather, our findings on *Presence* indicated that there were only small age-related differences, since most of the *Presence* factors showed no significant differences. In fact, the only significantly different *Presence* factor of Ecological Validity was higher in the *Older* group. These findings suggest that an older audience may have higher tolerance for shortcoming of the believability and realism of a virtual environment than a younger audience. For developers of VR applications this finding might be favorable as it may lower their development costs. Especially in the field of medical therapy and rehabilitation VR applications, higher Ecological Validity in older persons may pose the risk of a cognitive overload compared to younger people. This difference should also be considered in phobia treatment using VR, as fear-inducing situations might be perceived as more realistic by older patients. The significantly higher Ecological Validity values for the *Older* group in our study might partially be explained by their lesser VR experience and their assumed lesser experience with virtual worlds in general. Vice versa, Ecological Validity indicates here that younger people seem to be more critical regarding the authenticity or realness of the environment. One could therefore argue that more work has to be put into the creation of an improved virtual scenario when the target audience of the VR environment consists of predominately younger people. However, Kober [41] and our work have been able to show both that there is need for further research on age-related effects on *Presence* to gain reliable knowledge. Another consideration is that since most participants in the *Older* group had not been exposed to VR based scenarios for a significant duration of their lifespan, which was significantly different in the Younger group, it may affect their adaptability to *Presence* and *User Experience*; it may also limit, to some extent, their ability to become familiar with an alien technology. These considerations also put our study in a static context, reflecting the experience of today’s younger and older population. Likewise, in a few decades, the experience of a comparably older population may vary markedly from the contemporary experience of populations at different age groups.

In another study with a smaller number of participants (n = 20), Felnhofer et al. [40] investigated gender-related differences on *Presence* by giving a 5-minute speech in front of a virtual audience experienced with a Head-Mounted Display (HMD) and concluded that VR seems to be ‘made for males’ using the iGroup Presence Questionnaire (IPQ) [53]. Felnhofer et al. report a lower ‘Sense of being there’, ‘Spatial Presence’ and ‘Realness’ for women. Sagnier et al. [42] performed a study (n = 52) on gender-related difference in participants experienced with an HMD with a manual assembly task in the context of an aircraft using the Witmer & Singer presence questionnaire [54]. They report that ‘self-assessment of performance’ and ‘ability to act’ are lower for women. In contrast to Felnhofer et al. [40] and Sagnier et al. [42], we report no significant gender-related effects on *Presence*. Due to the different experimental tasks, the different presence questionnaires and VR technologies (HMDs vs. CAVE), these three studies and their contradicting findings are difficult to compare. However, there are four common points in the work of Felnhofer et al. and Sagnier et al. that are different from our presented work: (1) the experimental tasks were performed at one place and did not require the participant to move through the virtual environment: (2) the experimental tasks had serious consequential contexts; (3) the participants were only interacting with a virtual object; and (4) the participants could not see their real bodies. Neither Felnhofer et al. nor Sagnier et al. provided any information if the participants were presented with a virtual body or not. Since both studies also did not report on any kind of body tracking, we assume that, at best, the participants were presented with virtual hands. For our study, these four points were different: (1) moving through the virtual environment was a crucial part of the experimental task; (2) the geocaching game can be considered a leisure time and fun activity; (3) the participants used a real smart phone in context of the virtual environment; and (4) the participants were able to see their real bodies at all times. It would be too speculative to make assumptions on whether any of these four points are able to explain the different results found by Felnhofer et al., Sagnier et al., and in our study. However, future studies could investigate if there are circumstances where a VR experiences could lead to the differences in *Presence* based on gender. Furthermore, in the same study, Sagnier et al. [42] also investigated *User Experience* using the AttrakDiff2 [55] questionnaire. They found lower scores for women with ‘hedonic quality stimulation’, but not with ‘hedonic quality identification’ and ‘pragmatic quality’, which, in general, supports our results of not finding any differences *User Experience* or *Usability* based on gender. However, the deviating result for ‘hedonic quality stimulation’ calls for further investigation in future studies.

It seems highly relevant to find a conclusive answer to the question how age and gender influence *Presence* is crucial for developers of commercial VR applications and professional users (e.g., therapists) alike. The success of VR applications could be influenced negatively in case age- or gender-related adaptions are necessary for all users to fully enjoy them. In a worst case scenario, professional users including therapist might even improperly treat their patients due to age or gender related differences in the impact of the VR experience. Our results suggest that no gender aspects have to be considered by developers of commercial VR applications and professional users. However, in terms of age, the higher perceived Ecological Validity of a virtual experience must especially wary professional users not to overexcite users.

The results of this study for *User Experience* (UEQ) and *Usability* (SUS) of the *Younger* and *Older* groups were contrary. The UEQ showed higher results for the *Older* group, meaning that they liked the application better, which may have been related to the novelty of VR and geocaching. In contrast, the SUS yielded significantly higher scores in the *Younger* group. These finding for the UEQ and SUS may have resulted from the nature of the UEQ and SUS. The UEQ questionnaire asks on an abstract level for bipolar adjectives associated with the application. In contrast, the SUS consists of items that directly ask about the application, such as ‘I think I would like to use this system frequently’. Whilst the UEQ reflects the older participants’ subtle assessment of the application and their general enjoyment of the new experience, the SUS askes directly for their opinion. This difference is in contrast to the SUS findings that speak in favor of not using the application in the future. An alternative, speculative interpretation of the conflicting UEQ and SUS ratings could be that the older participants in general liked and enjoyed the geocaching game but did not see any real meaning in it.

In summary, the novel findings in this study are the following:

- In contrast to existing literature, a decrease was observed in *Presence* with age for the current population with no previous VR experience. Instead, only a significant difference for the factor ‘Ecological Validity’ was found, which was higher for the *Older* group.
- In contrast to existing literature, no gender-effect on *Presence* was seen. Furthermore, no gender-effect on *User Experience* and *Usability* was observed.
- Age was found to have contradicting effects on *Usability* (lower) and on two *User Experience* Factors.
- Future research should be directed to clarify the effects of age and gender on *Presence*. Further, the reasons for the contradicting results of age effect on *User Experience* and *Usability* should be investigated.

A number of limitations need to be addressed for this study. The first limitation is in regards to the mode of locomotion used for the geocaching scenario. A few participants struggled with controlling their movements in VR with the Microsoft Kinect sensor-based navigation method. Furthermore, there were known technical limitations with the sensor itself, regarding movement recognition. Both issues did not affect the results in previous studies using the same navigation method [30, 43] and could probably be solved in the future using a more stable tracking system. Second, some glass wearers interrupted the test for a few seconds when they re-adjusted the fit of their glasses and the VR-glasses that had to be worn additionally. Further, the Hawthorne effect might have influenced the results, as the users may have anticipated to be exposed to something novel and exciting, to the end that the participants rated their experience in favor of this anticipation. Lastly, our study must be seen as a snapshot in time, given that the general population might have gained their first experiences with VR that are not yet part of their everyday life. To investigate possible changes resulting from VR becoming more widespread, our study should be re-evaluated in 3-year intervals. Therefore, our work can only be seen as a starting point for long-term investigations on how the increasing exposure of VR affects *Presence* and its connection with *User Experience* and *Usability*.

Future studies should also use other *Presence*, *User Experience* and *Usability* questionnaires in conjunction with different study tasks to derive strongly reliable and generalizable statements. It would furthermore be very interesting to see if the found age-dependent difference for the *Presence* item of Ecological Validity would change if the participants were exposed to multiple environments over a longer period to compensate for different experience levels, possibly also using other VR devices like head mounted displays. Especially, the impact of technology affinity and a detailed differentiation of levels of VR-experience on *Presence* and its connection with *User Experience* and *Usability*, should be focused.

## 5 Conclusion

In this study, both age- and gender-related effects on *Presence*, *User Experience* and *Usability* in VR are jointly investigated for the first time. The body of literature investigating gender and age related is very limited and calls for further investigations. In contrast to existing literature, we could not prove gender-related differences on *Presence*, nor that *Presence* decreases with age. However, we present four discriminating factors of this work with the existing literature to further investigate possible gender related effects on *Presence*, *User Experience* and *Usability*. Our findings suggest that, for the most part, no major age- or gender-related differences exist on *Presence*. However, older participants seemed to find the VR environment more realistic than the younger participants. Further, no interaction effects were found, and only minor age- and gender-related influence on the results of the ITC-SOPI, UEQ and SUS questionnaires were found. The results for *User Experience* were higher for the older participants whilst for *Usability* the younger participants showed higher ratings.

## Supporting information

S1 Dataset Minimal dataset.csv contains the minimal dataset used for the statistical evaluation and for deriving Figs 3–5. (CSV)

## References

[1] Lui T-W, Piccoli G, Ives B. Marketing strategies in virtual worlds. SIGMIS Database. 2007; 38:77. doi: 10.1145/1314234.1314248

[2] Alcañiz M, Bigné E, Guixeres J. Virtual Reality in Marketing: A Framework, Review, and Research Agenda. Front Psychol. 2019; 10:1530. Epub 2019/07/05. doi: 10.3389/fpsyg.2019.01530 . 31333548 PMC6624736

[3] Regt A de, Plangger K, Barnes SJ. Virtual reality marketing and customer advocacy: Transforming experiences from story-telling to story-doing. Journal of Business Research. 2021; 136:513–22. doi: 10.1016/j.jbusres.2021.08.004

[4] Yung R, Khoo-Lattimore C, Potter LE. Virtual reality and tourism marketing: conceptualizing a framework on presence, emotion, and intention. Current Issues in Tourism. 2021; 24:1505–25. doi: 10.1080/13683500.2020.1820454

[5] Choi S, Jung K, Noh SD. Virtual reality applications in manufacturing industries. Past research, present findings, and future directions. Concurrent Engineering. 2014; 23:40–63. doi: 10.1177/1063293X14568814

[6] Silva RKJ de, Rupasinghe TD, Apeagyei P. A collaborative apparel new product development process model using virtual reality and augmented reality technologies as enablers. International Journal of Fashion Design, Technology and Education. 2019; 12:1–11. doi: 10.1080/17543266.2018.1462858

[7] Ciprian Firu A, Ion Tapîrdea A, Ioana Feier A, Drăghici G. Virtual reality in the automotive field in industry 4.0. Materials Today: Proceedings. 2021; 45:4177–82. doi: 10.1016/j.matpr.2020.12.037

[8] Aïm F, Lonjon G, Hannouche D, Nizard R. Effectiveness of Virtual Reality Training in Orthopaedic Surgery. Arthroscopy. 2016; 32:224–32. doi: 10.1016/j.arthro.2015.07.023 . 26412672

[9] Gavish N, Gutiérrez T, Webel S, Rodríguez J, Peveri M, Bockholt U, et al. Evaluating virtual reality and augmented reality training for industrial maintenance and assembly tasks. Interactive Learning Environments. 2013; 23:778–98. doi: 10.1080/10494820.2013.815221

[10] Tubelo RA, Branco VLC, Dahmer A, Samuel SMW, Collares FM. The influence of a learning object with virtual simulation for dentistry. A randomized controlled trial. Int J Med Inform. 2016; 85:68–75. doi: 10.1016/j.ijmedinf.2015.11.005 . 26601728

[11] Zahabi M, Abdul Razak AM. Adaptive virtual reality-based training: a systematic literature review and framework. Virtual Reality. 2020; 24:725–52. doi: 10.1007/s10055-020-00434-w

[12] Kaplan AD, Cruit J, Endsley M, Beers SM, Sawyer BD, Hancock PA. The Effects of Virtual Reality, Augmented Reality, and Mixed Reality as Training Enhancement Methods: A Meta-Analysis. Hum Factors. 2021; 63:706–26. Epub 2020/02/24. doi: 10.1177/0018720820904229 . 32091937

[13] Saposnik G. Virtual Reality in Stroke Rehabilitation. In: Ovbiagele B, editor. Ischemic Stroke Therapeutics. Cham: Springer International Publishing; 2016. pp. 225–33.

[14] Laver KE, George S, Thomas S, Deutsch JE, Crotty M. Virtual reality for stroke rehabilitation. Cochrane Database Syst Rev. 2015:CD008349. doi: 10.1002/14651858.CD008349.pub3 . 25927099 PMC6465102

[15] Gourlay D, Lun KC, Lee YN, Tay J. Virtual reality for relearning daily living skills. Int J Med Inform. 2000; 60:255–61. doi: 10.1016/s1386-5056(00)00100-3 11137470

[16] Beidel DC, Frueh BC, Neer SM, Bowers CA, Trachik B, Uhde TW, et al. Trauma management therapy with virtual-reality augmented exposure therapy for combat-related PTSD. A randomized controlled trial. J Anxiety Disord. 2019; 61:64–74. doi: 10.1016/j.janxdis.2017.08.005 . 28865911

[17] Rutkowski S, Kiper P, Cacciante L, Cieślik B, Mazurek J, Turolla A, et al. Use of virtual reality-based training in different fields of rehabilitation: A systematic review and meta-analysis. J Rehabil Med. 2020; 52:jrm00121. Epub 2020/11/19. doi: 10.2340/16501977-2755 . 33073855

[18] Xie M, Zhou K, Patro N, Chan T, Levin M, Gupta MK, et al. Virtual Reality for Vestibular Rehabilitation: A Systematic Review. Otol Neurotol. 2021; 42:967–77. doi: 10.1097/MAO.0000000000003155 . 33782257

[19] Nascimento AS, Fagundes CV, Mendes FADS, Leal JC. Effectiveness of Virtual Reality Rehabilitation in Persons with Multiple Sclerosis: A Systematic Review and Meta-analysis of Randomized Controlled Trials. Mult Scler Relat Disord. 2021; 54:103128. Epub 2021/07/09. doi: 10.1016/j.msard.2021.103128 . 34280679

[20] Valmaggia LR, Latif L, Kempton MJ, Rus-Calafell M. Virtual reality in the psychological treatment for mental health problems. An systematic review of recent evidence. Psychiatry Res. 2016; 236:189–95. doi: 10.1016/j.psychres.2016.01.015 . 26795129

[21] Parsons TD, Rizzo AA. Affective outcomes of virtual reality exposure therapy for anxiety and specific phobias. A meta-analysis. J Behav Ther Exp Psychiatry. 2008; 39:250–61. doi: 10.1016/j.jbtep.2007.07.007 . 17720136

[22] North MM, North SM. Virtual Reality Therapy for Treatment of Psychological Disorders. In: Maheu MM, Drude KP, Wright SD, editors. Career Paths in Telemental Health. Cham: Springer International Publishing; 2017. pp. 263–8.

[23] Karami B, Koushki R, Arabgol F, Rahmani M, Vahabie A-H. Effectiveness of Virtual/Augmented Reality-Based Therapeutic Interventions on Individuals With Autism Spectrum Disorder: A Comprehensive Meta-Analysis. Front Psychiatry. 2021; 12:665326. Epub 2021/06/23. doi: 10.3389/fpsyt.2021.665326 . 34248702 PMC8260941

[24] Skarbez R, Brooks JFP, Whitton MC. A Survey of Presence and Related Concepts. ACM Comput Surv. 2018; 50:1–39. doi: 10.1145/3134301

[25] ISO 9241–210. Ergonomics of human–system interaction–Part 210: Human-centred design for interactive systems. 2010. Ergonomics of human system interaction-Part 210: Human-centred design for interactive systems. Schweiz; 15.03.2010 [updated 2010 Mar 15].

[26] Kim YM, Rhiu I, Yun MH. A Systematic Review of a Virtual Reality System from the Perspective of User Experience. International Journal of Human-Computer Interaction. 2019; 189:1–18. doi: 10.1080/10447318.2019.1699746

[27] Mäkinen H, Haavisto E, Havola S, Koivisto J-M. User experiences of virtual reality technologies for healthcare in learning: an integrative review. Behaviour & Information Technology. 2022; 41:1–17. doi: 10.1080/0144929X.2020.1788162

[28] Xie B, Liu H, Alghofaili R, Zhang Y, Jiang Y, Lobo FD, et al. A Review on Virtual Reality Skill Training Applications. Front virtual real. 2021; 2. doi: 10.3389/frvir.2021.645153

[29] Huang H-M, Liaw S-S, Lai C-M. Exploring learner acceptance of the use of virtual reality in medical education. A case study of desktop and projection-based display systems. Interactive Learning Environments. 2016; 24:3–19. doi: 10.1080/10494820.2013.817436

[30] Brade J, Lorenz M, Busch M, Hammer N, Tscheligi M, Klimant P. Being there again–Presence in real and virtual environments and its relation to usability and user experience using a mobile navigation task. International Journal of Human-Computer Studies. 2017; 101:76–87. doi: 10.1016/j.ijhcs.2017.01.004

[31] Moffat SD, Resnick SM. Effects of age on virtual environment place navigation and allocentric cognitive mapping. Behavioral Neuroscience. 2002; 116:851–9. doi: 10.1037//0735-7044.116.5.851 12369805

[32] Moffat SD, Zonderman AB, Resnick SM. Age differences in spatial memory in a virtual environment navigation task. Neurobiology of Aging. 2001; 22:787–96. doi: 10.1016/s0197-4580(01)00251-2 11705638

[33] CUTMORE TR, HINE TJ, MABERLY KJ, LANGFORD NM, HAWGOOD G. Cognitive and gender factors influencing navigation in a virtual environment. International Journal of Human-Computer Studies. 2000; 53:223–49. doi: 10.1006/ijhc.2000.0389

[34] Driscoll I, Hamilton DA, Yeo RA, Brooks WM, Sutherland RJ. Virtual navigation in humans. The impact of age, sex, and hormones on place learning. Horm Behav. 2005; 47:326–35. doi: 10.1016/j.yhbeh.2004.11.013 . 15708762

[35] Lin J, Cao L, Li N. Assessing the influence of repeated exposures and mental stress on human wayfinding performance in indoor environments using virtual reality technology. Advanced Engineering Informatics. 2019; 39:53–61. doi: 10.1016/j.aei.2018.11.007

[36] Corriveau Lecavalier N, Ouellet É, Boller B, Belleville S. Use of immersive virtual reality to assess episodic memory: A validation study in older adults. Neuropsychol Rehabil. 2020; 30:462–80. Epub 2018/05/29. doi: 10.1080/09602011.2018.1477684 . 29807474

[37] Zibrek K, Niay B, Olivier A-H, Hoyet L, Pettre J, McDonnell R. The Effect of Gender and Attractiveness of Motion on Proximity in Virtual Reality. ACM Trans Appl Percept. 2020; 17:1–15. doi: 10.1145/341998534113222

[38] Schwind V, Knierim P, Tasci C, Franczak P, Haas N, Henze N. "These are not my hands!". In: Mark G, Fussell S, Lampe C, Schraefel M, Hourcade JP, et al., editors. Proceedings of the 2017 CHI Conference on Human Factors in Computing Systems. New York, NY, USA: ACM; 2017. pp. 1577–82.

[39] Peck TC, Sockol LE, Hancock SM. Mind the Gap: The Underrepresentation of Female Participants and Authors in Virtual Reality Research. IEEE Trans Vis Comput Graph. 2020; 26:1945–54. Epub 2020/02/13. doi: 10.1109/TVCG.2020.2973498 . 32070984

[40] Felnhofer A, Kothgassner OD, Beutl L, Hlavacs H, Kryspin-Exner I. Is Virtual Reality made for Men only? Exploring Gender Differences in the Sense of Presence. Proceedings of the International Society on presence research. 2012. pp. 103–12.

[41] Kober SE. Effects of Age on the Subjective Presence Experience in Virtual Reality. In: Felnhofer A, editor. Challenging presence. Proceedings of the International Society for Presence Research 15th International Conference on Presence. Wien: Facultas.WUV; 2014. pp. 149–157.

[42] Sagnier C, Loup-Escande E, Valléry G. Effects of Gender and Prior Experience in Immersive User Experience with Virtual Reality. In: Ahram T, Falcão C, editors. Advances in Usability and User Experience. Cham: Springer International Publishing; 2020. pp. 305–14.

[43] Lorenz M, Brade J, Diamond L, Sjölie D, Busch M, Tscheligi M, et al. Presence and User Experience in a Virtual Environment under the Influence of Ethanol. An Explorative Study. Sci Rep. 2018; 8:6407. doi: 10.1038/s41598-018-24453-5 . 29686255 PMC5913276

[44] Gates GJ. How Many People are Lesbian, Gay, Bisexual and Transgender?; 2011.

[45] Zwick S, Rauprich J. Actionbound. 2021 [cited 28 Jul 2021]. Available from: https://en.actionbound.com/.

[46] Cruz-Neira C, Sandin DJ, DeFanti TA, Kenyon RV, Hart JC. The CAVE. Audio visual experience automatic virtual environment. Commun ACM. 1992; 35:64–72. doi: 10.1145/129888.129892

[47] Cruz-Neira C, Sandin DJ, DeFanti TA. Surround-screen projection-based virtual reality. In: Whitton MC, editor. Proceedings of the 20th annual conference on Computer Graphics and Interactive Techniques—SIGGRAPH ’93. New York, New York, USA: ACM Press; 1993. pp. 135–42.

[48] Lorenz M, Busch M, Rentzos L, Tscheligi M, Klimant P, Frohlich P. I’m There! The influence of virtual reality and mixed reality environments combined with two different navigation methods on presence. In: Höllerer T, Interrante V, Lécuyer A, Swan JE II, editors. 2015 IEEE Virtual Reality (VR).; 2015. pp. 223–4.

[49] Lessiter J, Freeman J, Keogh E, Davidoff J. A Cross-Media Presence Questionnaire. The ITC-Sense of Presence Inventory. Presence: Teleoperators and Virtual Environments. 2001; 10:282–97. doi: 10.1162/105474601300343612

[50] Hassenzahl M, Tractinsky N. User experience—a research agenda. Behaviour & Information Technology. 2006; 25:91–7. doi: 10.1080/01449290500330331

[51] Laugwitz B, Held T, Schrepp M. Construction and Evaluation of a User Experience Questionnaire. In: Holzinger A, editor. HCI and Usability for Education and Work: 4th Symposium of the Workgroup Human-Computer Interaction and Usability Engineering of the Austrian Computer Society, USAB 2008, Graz, Austria, November 20–21, 2008. Proceedings. Berlin, Heidelberg: Springer Berlin Heidelberg; 2008. pp. 63–76.

[52] Brooke J. SUS-A quick and dirty usability scale. Usability Evaluation in Industry. 1996; 189:4–7.

[53] Schubert T, Friedmann F, Regenbrecht H. The Experience of Presence. Factor Analytic Insights. Presence: Teleoperators and Virtual Environments. 2001; 10:266–81. doi: 10.1162/105474601300343603

[54] Witmer BG, Singer MJ. Measuring Presence in Virtual Environments. A Presence Questionnaire. Presence: Teleoperators and Virtual Environments. 1998; 7:225–40. doi: 10.1162/105474698565686

[55] Hassenzahl M, Burmester M, Koller F. AttrakDiff: Ein Fragebogen zur Messung wahrgenommener hedonischer und pragmatischer Qualität. In: Szwillus G, Ziegler J, editors. Mensch & Computer 2003. Wiesbaden: Vieweg+Teubner Verlag; 2003. pp. 187–96.

